# Complete mitochondrial genome sequence of Japanese forest green tree frog (*Rhacophorus arboreus*)

**DOI:** 10.1080/23802359.2020.1820396

**Published:** 2020-09-16

**Authors:** Hidetoshi Inagaki, Yoshikazu Haramoto, Hiroshi Y. Kubota, Yasushi Shigeri

**Affiliations:** a Biomedical Research Institute, National Institute of Advanced Industrial Science and Technology (AIST), Tsukuba, Japan; b Cellular and Molecular Biotechnology Research Institute, National Institute of Advanced Industrial Science and Technology (AIST), Tsukuba, Japan; c Department of Zoology, Graduate School of Science, Kyoto University, Kyoto, Japan; d Department of Chemistry, Wakayama Medical University, Wakayama, Japan

**Keywords:** Mitochondrial genome, *Rhacophorus arboreus*

## Abstract

We determined the complete mitochondrial genome sequence of the Japanese forest green tree frog (*Rhacophorus arboreus*). The mitochondrial genome is 22,236 bp in length, which encodes 13 protein-coding genes, 2 rRNA, and 22 tRNA genes, and two control regions (D-loops). The whole gene arrangement of *R. arboreus* was the same as that of *Rhacophorus omeimontis* and *Rhacophorus schlegelii*.

The Japanese forest green tree frog (*Rhacophorus arboreus*) is a species endemic to Japan; it lives in mountain forests, and is famous for laying eggs on trees with a unique foam (Okada and Kawano 1924; Kusano et al. 2006). This species is classified as a Near-Threatened species in some local regions in Japan (Kaneko and Matsui 2004). To elucidate the phylogenetic relationship of this species in the genus *Rhacophorus,* we sequenced the complete mitochondrial genome of *R. arboreus.* Frogs were collected at Hieidaira, Otsu, Shiga, Japan (35°31,245′N, 135°827394′E) on 19 June 2019, since this species has not been protected in Shiga prefecture. Following the separation of mitochondria from the entire oviduct, the mitochondrial genome DNA was extracted. The mitochondrial genome DNA was deposited in Center for Molecular Biodiversity Research, National Museum of Nature and Science, Japan (NSMT-DNA 24269). A genome library was prepared using TruSeq DNA PCR-Free Library Prep Kit (Illumina, San Diego, CA, USA), and sequencing by NovaSeq6000 (Illumina) was carried out. Library preparation and sequencing were performed by Macrogen Japan (Kyoto, Japan). The *de novo* assembles were run by Velvet version 1.2.10 (Zerbino and Birney 2008). The coding regions were roughly predicted by MITOS Web Server (Bernt et al. 2013) and adjusted manually.

Due to the high similarity of two D-loops and short repeated sequences around them, the mitochondrial genome was assembled incompletely and was divided into four fragments. To fill the gap among the fragments, we assembled the fragments by PCR products generated from the mitochondrial genome DNA and seven primers (Table S1). PCR fragments were cloned into pCR2.1-TOPO (Thermo Fisher Scientific, Waltham, MA, USA), and sequenced by ABI 3500 Genetic Analyzer (Thermo Fisher Scientific).

The mitochondrial genome of *R. arboreus* is 22,236 bp long and composed from 13 protein-coding, two rRNA, and 22 tRNA genes. The whole mitochondrial genome sequence of *R. arboreus,* which was deposited in the DNA Data Bank of Japan (DDBJ) under accession no. LC565708, shows high identity with those of *Rhacophorus dennysi* (KM035412, 84.5% identity)*, Rhacophorus omeimontis* (MN427892, 88.7% identity), and *Rhacophorus schlegelii* (AB202078, 88.6% identity). Moreover, the duplication of the D-loop and the gene arrangement of *R. arboreus* are the same as those of *R. omeimontis* (Fu et al. 2020) and *R. schlegelii* (Sano et al. 2005). Despite the high sequence identity between *R. arboreus* and *R. dennysi* whole mitochondrial genome sequences, *R. dennysi* has only one D-loop (Huang et al. 2016).

In order to elucidate the phylogenetic relationship of the genus *Rhacophorus*, we aligned the mitochondrial 12S rRNA, tRNA-Val, 16S rRNA, and COI sequences of 15 *Rhacophorus* species by ClustalW, and performed a maximum-likelihood (ML) analysis to generate a phylogenetic tree. Because the sequence of *R. dennysi* in the previous study (LC010575, 461 bp) lacks 12S rRNA and tRNA-Val, we replaced the corresponding sequences from the complete mitochondrial genome sequence of *R. dennysi* (KM035412, 1901 bp). As shown in Figure 1, the phylogenic relationship among *Rhacophorus* species in our study is similar to the relationship in the previous study (Matsui et al. 2019), except for *R. dennysi.*

**Figure 1.**
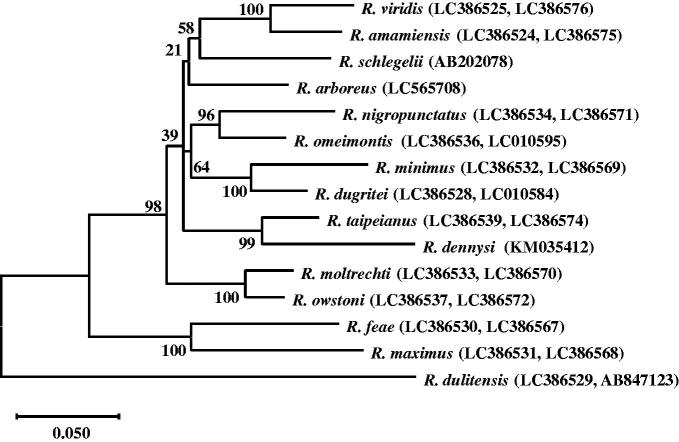
Phylogenetic relationship in the genus *Rhacophorus*. We aligned the sequences by ClustalW and constructed a maximum-likelihood (ML) phylogenetic tree based on the mitochondrial 12S rRNA, tRNA-Val, 16S rRNA, and COI sequences of 15 species by MEGA version X (Kumar et al. 2018). Most of the mitochondrial genome sequences are reported in the previous study (Matsui et al. 2019), but the sequences of *R. dennysi*, *R. schlegelii*, and *R. arboreus* were replaced with the corresponding sequences from the whole mitochondrial genomes. DDBJ accession numbers are noted in brackets. The values of the branches are bootstrap values (1000 replicates).

## Supplementary Material

Supplemental Material

## Data Availability

The data that support the findings of this study are openly available in DDBJ (accession no. LC565708) at https://www.ddbj.nig.ac.jp.
